# Governmental Inter-sectoral Strategies to Prevent and Control COVID-19 in a Megacity: A Policy Brief From Shanghai, China

**DOI:** 10.3389/fpubh.2022.764847

**Published:** 2022-02-08

**Authors:** Xiaolin He, Ping Jiang, Qiong Wu, Xiaobin Lai, Yan Liang

**Affiliations:** ^1^Research Center for Urban Society, Shanghai Administration Institute, Shanghai, China; ^2^Department of Public Health, Shanghai Municipal Health Commission of Changning District, Shanghai, China; ^3^School of Nursing, Fudan University, Shanghai, China

**Keywords:** government, strategies, COVID-19, prevention, policy, inter-sectoral

## Abstract

This policy brief aims to help policymakers develop inter-sectoral interventions in megacities to prevent and control COVID-19. Based on the case of Changning District in Shanghai, China, several policy options are identified. The guiding principles include ensuring a coordinated national response (i.e., moderation is required in epidemic prevention and control); making science-based, precise, and differentiated epidemic control strategies; and establishing a joint prevention and control mechanism. Policy tools include localized management, closed-loop management, community grid management, digital management, and sub-population management. There is no “one size fits all” policy; however, it will be helpful to learn by trial and error through on-the-ground experience with minimal information in real time.

## Introduction

The management of the COVID-19 crisis is a multi-level governance issue (1). Moreover, how governments deal with the COVID-19 crisis is becoming a major research stream worldwide (2). Different national and local governments have taken widely differing strategies (3) in pandemic response management owing to the differences in existing political structures and dynamics (4). Previous research has found that sub-national contributions to policy are more important than national-level policies (5), and it is crucial to study regional variations to unpack the different roles of and interactions between political culture, public policies, and citizens' level of compliance (6). It is suggested that, while the distribution of authority between central and regional governments matters, territorial policy dynamics (such as hierarchical, competitive, cooperative or mixed) are even more important in driving multi-level responses to the emergency (7). Meanwhile, the role of cities is also important as cities are the first responders to pandemics (8).

Government inter-sectoral strategies are required to address the challenges of the public health crisis (9), and an inter-sectoral action has been taken in many public-health-related fields, such as tobacco control (10), child safety (11), and malaria control (12). However, we know little about how governments take inter-sectoral strategies in responding to and handling COVID-19 appropriately, especially in megacities. Although some core strategic policy tools, such as testing, tracing, social distancing, and early preparation (3, 8), have been recommended, we still lack knowledge of appropriate inter-sectoral collaboration to improve the coordination of cross-related institutions in the prevention and control of COVID-19.

**Figure 1:**
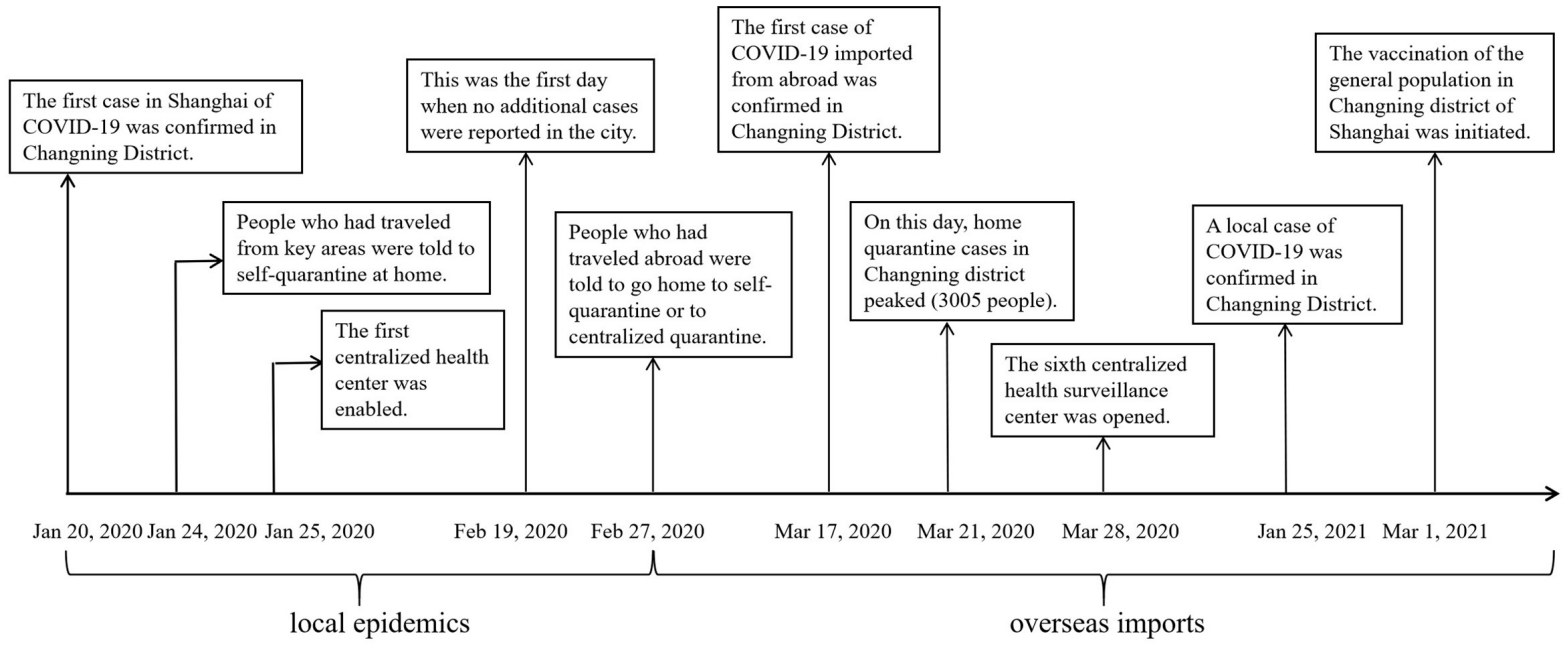
Timeline of the principal events for prevent and control COVID-19 in Changning district, Shanghai.

Shanghai is one of the biggest, most populated, most international cities in China. It is also a role model for other major cities and provinces in China in the prevention and control of COVID-19 (13). We chose Changning district in Shanghai as an observation case to provide an illustration of government's inter-sectoral strategies in the prevention and control of COVID-19 at the city, district, and community levels. Changning District, one of the 16 districts in Shanghai, located in the urban area of the city, is one of the most international districts, with one-fifth of residents being permanent foreigners and one-third being foreign consulates of Shanghai. Changning District has a permanent resident population of 693 thousand in 2020. The mean age of the permanent resident population is 44.6, and 45.24% of them have an educational level of college or over. Changning District's per capita GDP is about 35,000 USD and per capita disposable income is about 12500 USD in 2020. Changning District has been facing dual pressures of local epidemics and overseas imports. It has also taken initiatives in the prevention and control of COVID-19, such as digital governance. A timeline of the principal events for the prevention and control of COVID-19 in Changning District (Figure 1) will help us understand the context of policy options.

The COVID-19 pandemic has posed an enormous challenge to the government's decision-making because almost everything about the disease has been startlingly “novel,” so there has been no common agreement on the government's inter-sectoral strategies. Thus, it has been necessary to learn through on-the-ground experience and applied research. This policy brief seeks to briefly describe the policy options around government's inter-sectoral strategies to prevent and control COVID-19 in Changning District in Shanghai and provide actionable recommendations so as to inspire future policy and practice during this global public health emergency and beyond.

## Policy Options and Implications

### Method

We used an embedded qualitative single case study methodology to understand policy development and implementation (14). A wide range of stakeholders were included in in-depth interviews, group discussions, and observations, including senior decision makers, policy advisors, healthcare providers, and community officers. Publicly available documents and media articles were also reviewed. A thematic framework analysis was used for data analysis, which involved the processes of familiarizing, identifying a thematic framework, indexing, charting, mapping, and interpretation (15). Table 1 displays an overview about data collection methods and results presentation.

**Table 1:** An overview about data collection methods and results presentation.

**Method**	**Subject**	**Descriptive results shown in figures**			
		**Figure 1**	**Figure 2**	**Figure 3**	**Figure 4**
In-depth interviews	Senior decision makers at municipal and district levels; policy advisors; Health Commission officers; street officers	×	×	×	×
Focus group discussions	Healthcare providers; community officers		×		
Observation	Emergence response to local case				×
Document review	Policy documents	×	×	×	×

### Findings

#### Overall Strategies

**Figure 2:**
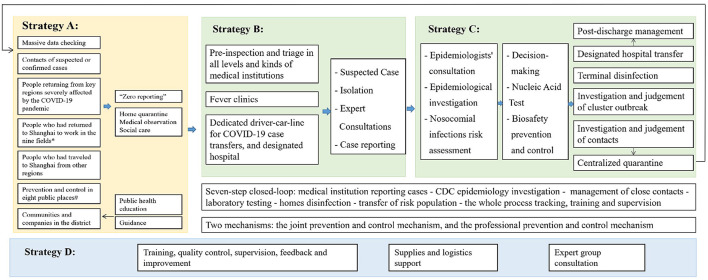
The overall strategies. Strategy A: reduce the overall cases; Strategy B: treat current cases; Strategy C: control the new confirmed cases; Strategy D: perform quality assurance; *The nine fields include: education, property management, logistics, public transportation, medical services, home services, nursing, labor-intensive plants, and companies; ^#^The eight public places include: residential quarters, neighborhood committees, farmers' markets and shops along the street, enterprise work resumption, office buildings, shopping malls, supermarkets, restaurants, and hotels. The yellow area represents joint prevention and control by the government; the green area displays the combination of medical treatment and prevention.

Figure 2 shows the overall strategies of Changning District in the prevention and control of COVID-19. Four main strategies were adopted: (A) Reducing the overall cases by focusing on key groups, key public places, and key fields. Measures taken included a reporting system, quarantine reinforcement, public health education, and guidance. (B) Treating the current cases by standardized workflows. (C) Controlling the new confirmed cases by investigation, scientific judgement, and closed-loop management. (D) Performing quality assurance through training, supervision, feedback and improvement, and logistics and expert support.

#### Government's Inter-sectoral Coordination

**Figure 3:**
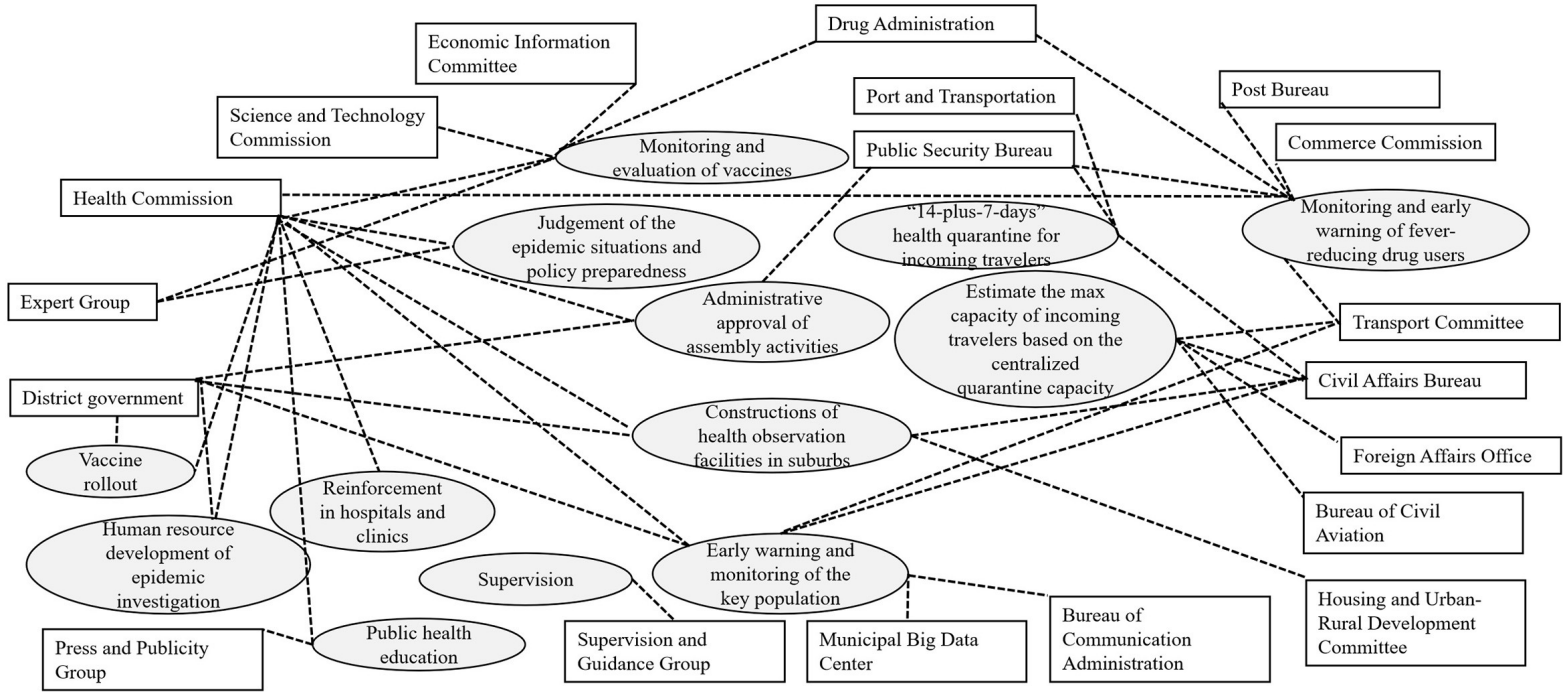
Government's inter-sectoral coordination.

Figure 3 displays the government's inter-sectoral coordination in the prevention and control of COVID-19. The joint prevention and control include judgment of epidemic situations and policy preparedness, monitoring and evaluation of vaccine, “14-plus-7-days” health quarantine for incoming travelers, and early warning and monitoring of the key population (the gray ovals in Figure 3). Almost all of them involved multiple agencies (the rectangles in Figure 3).

#### Supportive Information System

A government inter-sectoral data aggregation and visualization system was developed in Changning District to support the joint prevention and control of COVID-19. The system was based on the Shanghai municipal government's management platform and combined data from the city operation management network, the geographic information system (GIS), and the E-government WeChat system. This system aggregated personnel, resources, and task-related data to provide a “one-stop shop” for COVID-related information and enable real-time surveillance.

#### Closed-Loop Management

A closed-loop management model was developed to ensure a seamless and hermetic process for managing the quarantine and monitoring of travelers arriving in Shanghai, China. When international travelers arrived in Shanghai, they would be picked up by corresponding regional work groups according to their addresses, and placed in centralized quarantine for 14 days followed by home quarantine for 7 days. During their centralized and home quarantine, they would be asked to sign a quarantine promise letter. Healthcare workers and staff from neighborhood committees would provide medical observation and assistance. At least six nucleic acid tests would be performed during the 21 days.

#### Emergence Response to Reemergent Local Outbreak

**Figure 4:**
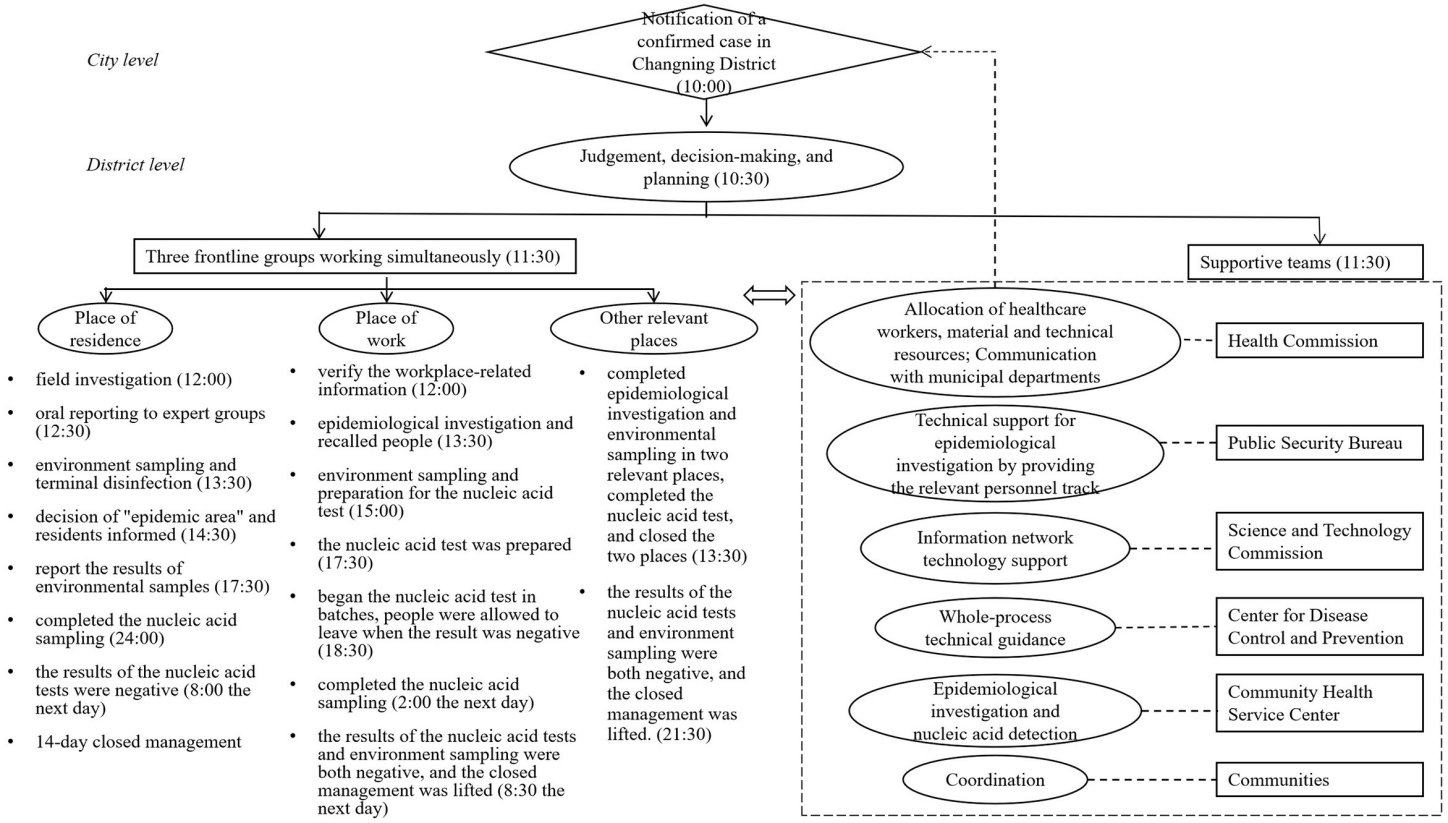
Map of implementation of the emergence response to reemergent local outbreak.

We took a local case in Changning District as an example. At 10 AM on January 25, 2021, a notification from the municipal was received by Changning District, indicating that, during the regular nucleic acid screening in hospitals, a case was confirmed to live and work in Changning District. The COVID-19 Prevention and Control Office of Changning District organized a meeting with the relevant agencies, and an action plan was made quickly based on scientific judgment. Six supportive teams and three frontline groups went to work simultaneously and completed this emergency response at 8 AM on January 26 to get the related risks under control. Figure 4 displays the map of implementation of the emergence response to a reemergent local outbreak. We learned the following about this emergency response: epidemiological investigation was the foundation, technical and coordinated teams provided support, action was taken at the beginning to handle the crisis, and plans were adjusted correspondingly according to the real-time updates of the investigation.

#### Vaccine Rollout

The vaccination of the general population in Changning District of Shanghai was initiated on March 1, 2021. By June 9, 2021, more than 1 million COVID-19 vaccination doses were administered in total. Comprehensive efforts were made to improve the population's vaccination coverage. In addition to the free vaccination policy, mobilization measures and the convenience of vaccination encouraged individuals to be vaccinated.

A multiple-mobilization strategy was adopted. *Community and company-based mobilization*: The vaccination teams reached out to every community and every company in the district to investigate and provide information and encourage individuals who were uncertain about vaccination to choose to be vaccinated. *Classified mobilization*: The Market Supervision Bureau focused on the owners and workers of shops along streets, and once vaccinated, the shop could be identified by a smiling visual identifier on the store front, and the education committee launched a “Little Hand in Big Hand” project to encourage family members of students to choose to be vaccinated. *Accessible vaccination opportunities*: More healthcare workers and more flexible vaccination time and locations were provided to give more workers access to vaccination. *Supervision*: Vaccination supervision was implemented to give continuous feedback and improvements.

### Actionable Recommendations

Based on the stakeholder perspectives, we make the following recommendations.

#### Guiding Principles

The policy should incorporate the following guiding principles. First, it should ensure a coordinated national response. The COVID-19 pandemic could continue for a longer period. With reemergent local outbreaks in different cities/areas, the city-level coordination needs to adjust with the national response. For example, in China, the green (health) code, yellow code, and red code are used to employ classified management of individuals from low-, medium-, and high-risk regions, respectively. Second, moderation is required in epidemic prevention and control to develop science-based, precise, and differentiated epidemic control strategies. Third, the policy should establish a joint prevention and control mechanism. Under the guidance of the Joint Prevention and Control Mechanism of the State Council, at the city level, work teams should cover epidemic prevention and control, medical treatment, scientific research, publicity, foreign affairs, logistics support, and work at the front line. They require strong synergy against the epidemic through close collaboration.

#### Policy Tools for Improving Government's Inter-sectoral Action

Several policy tools for improving government's inter-sectoral action are necessary. *Localized management:* Every district, street, and community must take responsibility. For example, the district takes responsible for the general implementation of national and municipal policies, involving action plan making, the breakdown of responsibilities, and explanation of quality target and the specification of controlling methods. The street takes responsible for the task decomposition and comprehensive resource allocation, coordination and combination. The community takes responsible for implementation of specific tasks. *Closed-loop management:* The whole process of management should be seamless and easily traced. *Community grid management:* A community-based grid should be established, and relevant personnel, resources, and tasks should be covered to form a matrix of communities. *Digital management:* We should rely on information technology to establish a platform to support data exchange, sharing, and coordination among inter-sectoral government to support governments' public health surveillance efforts. For example, to develop a system that aggregated personnel-, resource-, and task-related data to provide a one-stop source for COVID-related information, enabling real-time surveillance and facilitating the joint prevention and control of COVID-19 in a megacity. *Sub-population management:* There is a need to differentiate policies based on individual level characteristics to facilitate more tailored interventions. For example, for the elderly people, public authorities should consider their attitudes and compliance toward preventive measures (16), to make strategy to shield vulnerable population groups while keeping society as open as possible (17).

## Conclusions

The COVID-19 pandemic has posed new challenges for government management in megacities. It is necessary to develop government inter-sectoral strategies to prevent and control this pandemic. There is no specific “one size fits all” policy; however, it will be helpful to learn by trial and error through on-the-ground experience with minimal information in real time. Some guiding principles and policy tools were identified in this study to inspire future research, policy, and practice so as to support government emergency response.

## Author Contributions

XH, XL, and YL led the writing. All authors contributed to the conception and design of the article, manuscript revision, read, and approved the submitted version.

## Funding

This work was supported by Major Project of the Three-Year Action Plan (2020–2022) for Strengthening Public Health in Shanghai (GWV-13). The funder had no role in study design, data collection and analysis, decision to publish or preparation of the manuscript.

## Conflict of Interest

The authors declare that the research was conducted in the absence of any commercial or financial relationships that could be construed as a potential conflict of interest.

## Publisher's Note

All claims expressed in this article are solely those of the authors and do not necessarily represent those of their affiliated organizations, or those of the publisher, the editors and the reviewers. Any product that may be evaluated in this article, or claim that may be made by its manufacturer, is not guaranteed or endorsed by the publisher.
